# OrbiFragsNets. A tool for automatic annotation of orbitrap MS2 spectra using networks grade as selection criteria

**DOI:** 10.1016/j.mex.2023.102257

**Published:** 2023-06-14

**Authors:** Edwin Chingate, Jörg E. Drewes, María José Farré, Uwe Hübner

**Affiliations:** a Chair of Urban Water Systems Engineering, Technical University of Munich, Am Coulombwall 3, Garching 85748, Germany; b Catalan Institute for Water Research, Emili Grahit 101, Girona 17003, Spain; c Universitat de Girona, Girona, Spain

**Keywords:** Chemical consistency, Mass spectrometry, Orbitrap, Fragments networks, *OrbiFragsNets*

## Abstract

We introduce *OrbiFragsNets*, a tool for automatic annotation of MS2 spectra generated by Orbitrap instruments, as well as the concepts of chemical consistency and fragments networks. *OrbiFragsNets* takes advantage of the specific confidence interval for each peak in every MS2 spectrum, which is an unclear idea across the high-resolution mass spectrometry literature. The spectrum annotations are expressed as fragments networks, a set of networks with the possible combinations of annotations for the fragments. The model behind *OrbiFragsNets* is briefly described here and explained in detail in the constantly updated manual available in the GitHub repository. This new approach in MS2 spectrum *de novo* automatic annotation proved to perform as good as well established tools such as RMassBank and SIRIUS.

• A new approach on automatic annotation of Orbitrap MS2 spectra is introduced.
• Possible spectrum annotation are described as independent consistent networks, with annotations for each fragment as nodes, and annotations for the mass difference between fragments as edges.
• Annotation process is described as the selection of the most connected fragments network.

## Graphical abstract

**Figure:**
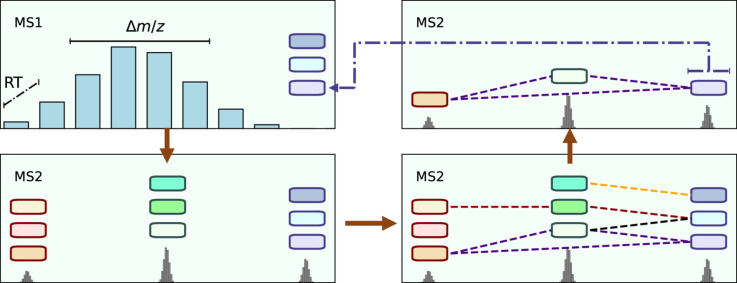

Specifications table

**Table:** 

Subject area:	Chemistry
More specific subject area:	*High-resolution mass spectrometry data analysis*
Name of your method:	*OrbiFragsNets*
Name and reference of original method:	N.A.
Resource availability:	https://github.com/EdwinChingate/OrbiFragsNets

## Introduction

Strong interest in new chemicals is shown by constantly growing public mass spectra databases, such as the MassBank [1] and the Global Natural Products Social Molecular Networking (GNPS) [2], as well as all the effort being spent on metabolomics [3] and natural products research [4]. The scientific community is now uncovering the novel biological and environmental effects caused by those unknown substances.

High-resolution mass spectrometry (HRMS), coupled with liquid chromatography (LC) or gas chromatography (GC), is a cutting-edge technology able to detect thousands of substances within a couple of minutes [5]. It is now essential for many scientific disciplines, from organic synthesis [5] to environmental sciences [6,7].

Instruments such as the Fourier-transform ion cyclotron resonance mass spectrometer (FTMS) [5] and Orbitrap [8] use the current image generated by ions inside an ion trap to determine a highly accurate mass-to-charge ratio (*m/z*) for MS2 product ions. Those fragmentation patterns might be informative enough to discover new molecules. However, the tricky relationship between the fragments and the chemical structure requires more research [9].

The current benchmark for elucidating the structure of small molecules (<1000 Da) is nuclear magnetic resonance (NMR) spectroscopy [[10], [11], [12]]. Nevertheless, NMR spectroscopy is extremely sensitive to impurities [12]. Purification makes the analysis with NMR spectroscopy challenging, expensive, time-consuming, and almost impossible to perform with complex mixtures of trace organic chemicals [13]. LC—HRMS, unlike NMR spectroscopy, detects individual molecules in complex mixtures using minor preliminary treatment steps, but requires a deeper data analysis.

Schymanski et al. [14] defined five confidence levels in identifying small molecules with HRMS. Level 1 is a confirmed structure. It requires the MS2 spectrum and retention time to match with standards in the same laboratory. Level 2 refers to a probable structure. It needs an MS2 spectrum match with a database. In level 3, the MS2 spectrum is ambiguous regarding isomers, as well as in level 4, where the confidence is just sufficient to define the molecular formula. Finally, reporting accurate mass is enough for lowest level of confidence, level 5.

The confidence in this classification system relies on data analysis and additional experiments after the HRMS analysis. With HRMS, data analysis conveys from level 5 to level 2, from cleaning the spectrum to comparing with databases. HRMS data is complicated to process, and different software alternatives disagree on crucial steps such as feature detection [15]. Annotation links the estimated *m/z* with a chemical formula. It increases the spectrum quality, facilitates interpretation [16], and makes comparing with databases more accessible [17].

There are several software alternatives for the automatic annotation of MS2 spectra. The RMassBank [16] and SIRIUS [18] are among the most popular. While the MassBank requires the RMassBank formatting for uploading spectra [[1], [16]], users prefer SIRIUS [18] after its compatibility with other tools such as MzMine [19], openms [20], and GNPS [2].

The RMassBank ranks the possible annotations for the peaks with their frequency appearance across different spectra on different adducts. In contrast, machine learning and comparison with databases define the annotations in SIRIUS. Both alternatives rely on data outside the target spectrum and reject possible annotations with a mass error. The user defines the mass error as a parameter, forcing certain accuracy on the *m/z* estimation, but the statistical treatment of experimental data would yield a better result [[7], [21]].

According to Brenton and Godfrey [21] an excessive report of long numbers as significant digits in the HRMS literature reflects an inappropriate use of concepts such as accurate mass, exact mass, and uncertainty. The massive amount of data generated on a single HRMS run demands automatic approaches and clarity in those concepts [[22], [23]].

**Fig. 1:**
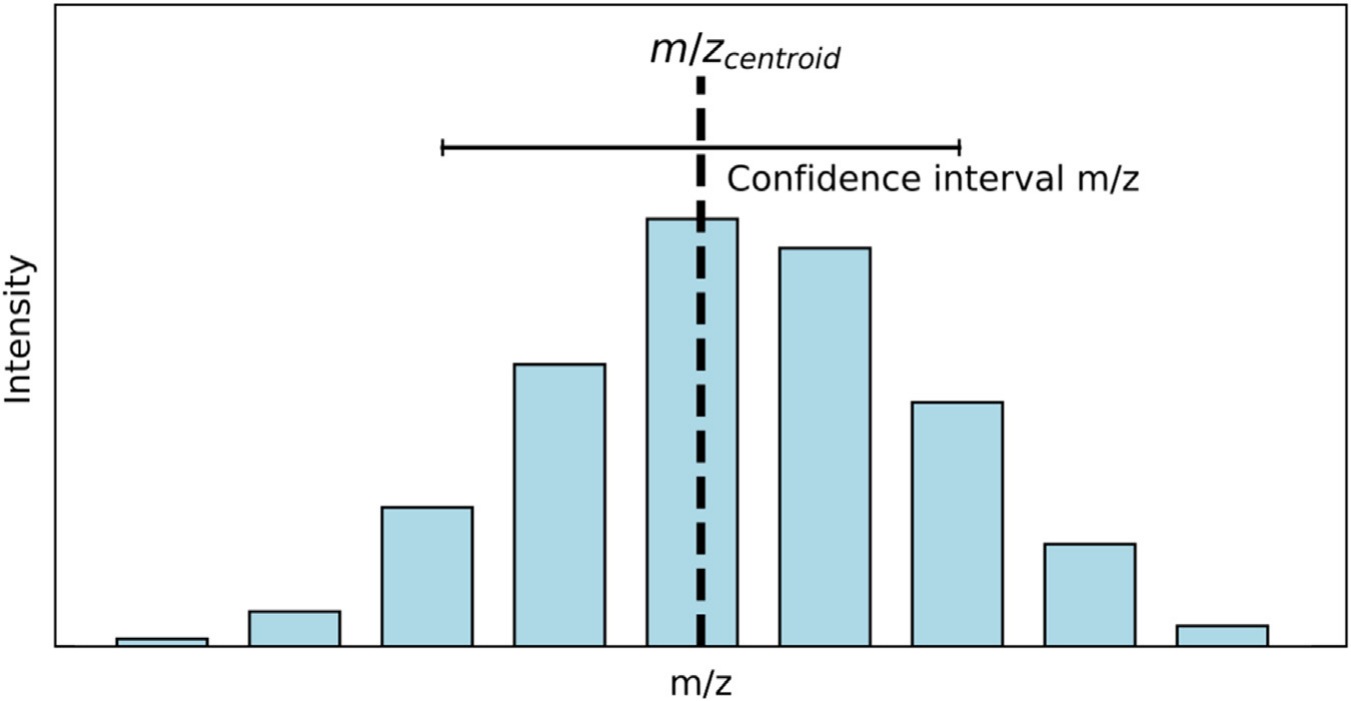
*m/z* distribution and *m/z*_centroid_. The centroiding process reduces the amount of data to handle, but the information on distribution and quality is lost.

Brenton and Godfrey [21] and Marty [23] recommend statistical evaluation of the *m/z* estimations across multiple measurements. However, every *m/z* peak in the Orbitrap data implies dynamic measurements [8]. The rawest data in an Orbitrap instrument is the image current generated by several ions in the ion trap for a certain period of time [8]. Then with the fast-Fourier transformation, data is converted into a distribution of frequencies related to the *m/z* for every ion [8]. This distribution of *m/z* resembles the peaks in the MS2 spectrum (Fig. 1) and is subject to statistical treatment by itself.

Unfortunately, this data distribution is lost while centroiding, as the first step in any workflow for HRMS data processing. This process aims to make the data analysis more manageable, but it sacrifices the uncertainty that is key for later tasks such as annotation (Fig. 1).

This article describes the basic data flow and usage for OrbiFragsNets, our software for the automatic annotation of DDMS2 (data dependent MS2) Orbitrap data. We expanded the concept by Brenton and Godfrey's [21] estimating confidence intervals on the raw data from only one spectrum. Then, we used the specific uncertainty for every peak to generate and filtrate the chemical space [22] of possible annotations. We employed networks theory to represent the different annotations on the MS2 spectrum as networks. Finally, we introduced the concept of chemical consistency, expressed it as the edges of the fragments networks, and used it to rank and select the best annotation. OrbiFragsNets aims to be an offline method for the automatic annotation of MS2 spectra generated by Orbitrap instruments.

### Model description

**Fig. 2:**
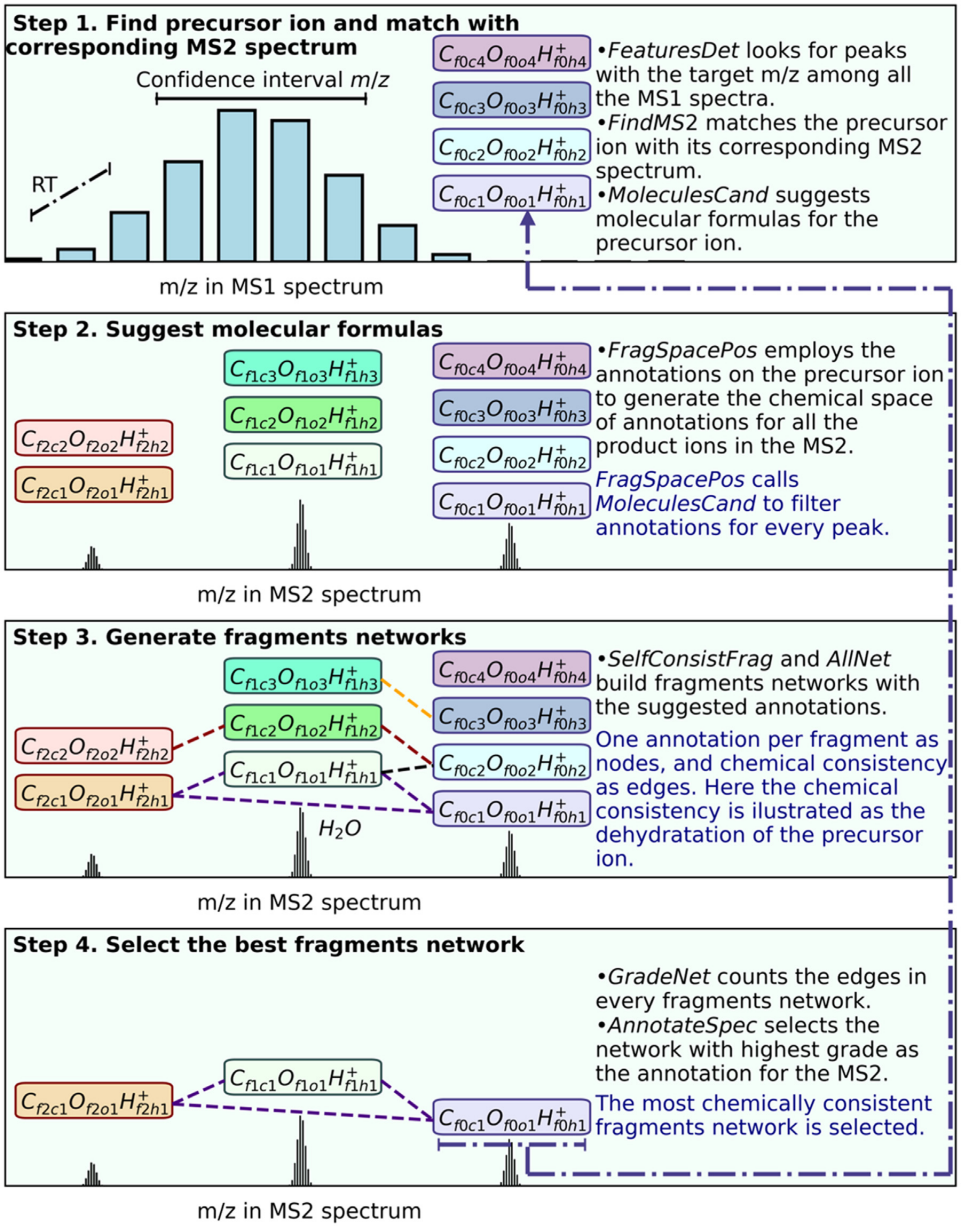
Internal workflow on the automatic annotation of MS2 spectra by *OrbiFragsNets. OrbiFragsNets* uses information the MS1 spectrum to suggest annotations (colorful boxes) on the target *m/z* and define a chemical space of annotations for the peaks in the MS2 spectrum. Then all the possible set of annotations are expressed as fragments networks, where nodes correspond with the annotations (colorful boxes) and the edges (broken lines connecting colorful boxes) with the chemical consistency. Finally, the most chemically consistent fragments network is selected as the annotation for the whole MS2 spectrum as well as the best molecular formula for the precursor ion.

*OrbiFragsNets* is written in Python3 and it can be executed from the any Linux terminal or a Jupyter-notebook. All the functions and the recommend Jupyter-notebook are available in the GitHub repository (https://github.com/EdwinChingate/OrbiFragsNets). The following paragraphs and Fig. 2 describe the model and data flow inside *OrbiFragsNets,* but the reader can find a detailed description of all the functions in the repository manual.

### Model assumptions and considerations

i. Every signal in the raw data is treated as a single data-point
ii. *m/z* for each peak follows a normal distribution. *OrbiFragsNets* confirms it with a Shapiro-test.
iii. The fragmentation process is always a decomposition reaction, from an ion into another ion and a neutral molecule (equation 1). The MS2 spectrum contains the signals from both ions and the *m/z* difference corresponds with the neutral molecule *m/z.OrbiFragsNets* describes it as a vector equation (equation 2). In the following sections this assumption will be refereed as “chemical consistency”.
  (1) $$C_{f0c}O_{f0o}H_{f0h}^{+}\;\to C_{f1c}O_{f1o}H_{f1h}^{++}C_{c}O_{o}H_{h}$$
  (2) (f0c,f0o,f0h)=(f1c,f1o,f1h)+(c,o,h)
iv. Each peak in the MS1 spectrum and the MS2 spectrum corresponds with a unique fragment and have a unique annotation. This assumption is easy to achieve with DDA, as it requires filtering the precursor ions in the MS1, prior to fragmentation.
v. Only molecules with one charge are considered in the chemical space for annotation.
vi. Only the product ions smaller than the precursor ion are consider as part of the MS2 spectrum.
vii. *OrbiFragsNets’* annotates using only the most abundant isotopes for the elements potassium, sodium, carbon, chlorine, sulfur, phosphorus, fluor, oxygen, nitrogen, and hydrogen; and the 13, and 34 isotopes for carbon, and sulfur, respectively.

Fig. 2 schematically illustrates our automatic annotation approach. Every step summarizes multiple functions in OrbiFragsNets. A fragments network will have as many nodes as peaks in the MS2 spectrum, examples in Tables 3 and 4 contain 16 and 18 fragments, respectively. The maximum number of edges in a n-nodes network is n*(n-1)/2, in case all fragments in the network are consistent between them. OrbiFragsNets generates as many networks as combinations of annotations for the individual fragments. For example, five fragments with five candidates for annotation will generate up to 3125 networks.

## *OrbiFragsNets* installation and usage

### Installation

All functions are written in *Python3* (https://www.python.org/) and we recommend to use the provided *Jupyter-notebook* (https://jupyter.org/) for execution. *OrbiFragsNets* uses the *Python* libraries *Numpy (*https://numpy.org/) and *Pandas* (https://pandas.pydata.org/) for data manipulation; *scipy* (https://scipy.org/) for statistical analysis; *IPython* (https://ipython.org/) and tabulate (https://pypi.org/project/tabulate/) for data visualization; *datetime* and *os* for files manipulation; and finally *Pyopenms* (https://pyopenms.readthedocs.io/en/latest/, [20]) for manipulation of the mass spectra files. Make sure to install the required libraries before proceeding with the following steps.

i. Open the GitHub repository *OrbiFragsNets* (https://github.com/EdwinChingate/OrbiFragsNets).
ii. Click the “Code” button, and select “Download ZIP”.
iii. Extract the content in your favorite folder.
iv. Confirm the existence of the file *OrbiFragsNets_Notebook.ipynb*, as well as, the folders ‘Data’, ‘Functions’, and ‘Parameters’.

### Usage

The process in Fig. 2 is fully automatic and the user of just needs to prepare the data in the right format (.mzML), charge it into the program and execute the function *OrbiFragsNets.* Beyond the following tutorial for annotation of a MS2 spectrum with a known *m/z*, all functions in *OrbiFragsNets* can be used independently, and adapted to scan a full data-set of unknown substances.

i. Get your raw data (.raw) from your experiment into your data analysis computer.
ii. Use *ProteoWizard msConvert* to convert your files into the .mzML format. Adusumilli and Mallick [24] already gave a detailed description of this step. Keep your data as raw as possible, in profile mode; avoid using filters in msConvert. Here is an example for the conversion from the linux terminal: ‘*msconvert "$file" –mzML*’
iii. Move your .mzML files to the folder ‘Data’ inside your working folder.
iv. Open your *Jupyter-notebook OrbiFragsNets_Notebook.ipynb*.
v. Execute the cell “Charge libraries”.
vi. In the cell “Charge your data file”, assign the name of the file that you want to analyse (.mzML) to the string variable ‘DataSetName’ and execute the function *ChargeDataSet*. Your file will be stored in the variable *DataSet*.
vii. Open the file *MaxAtomicSubscripts.csv*, inside the folder Parameters, with a text editor such as *gedit* in Ubuntu, or any spreadsheet software such as gnumeric or calc from open office, while making sure to keep the extension as .csv. Define the maximum number of atoms you expect in your molecule for every element or high values (>50) while sacrificing some seconds on the execution of *MoleculesCand*.
viii. Open the file *ParametersTable.csv*, the same way as *MaxAtomicSubscripts.csv*, and define your desired parameters. The repository manual provides a detailed description on each parameter, but the most relevant is the *NoiseTresInt*. Data from Orbitrap instruments is robust regarding noise signals after the clustering and the statistical test, but *NoiseTresInt* strongly affects the execution time in the function *NumpyMSPeaksIdentification*.
ix. Define the *m/z* for your target molecule as the protonated adduct (+*H*^+^), and execute the function *OrbiFragsNets.* Or as an anion and change the value for the parameter ‘ionization’ in the *ParametersTable.csv* from ‘+’ to ‘-’.
x. Use the following cell “ShowDF” to visualize your results table.
xi. Export your table as an .xlsx or .csv file in the cell “Export results”. The columns ‘Predicted *m/z* (Da)’ and ‘Relative intensity (%)’ can be used searching for similar spectra in the MSBank, as well, as the column ‘Molecular formula’ would assist its interpretation.

Table 1 displays the automatic annotation, for our own sulfamethoxazole Orbitrap analysis, by *OrbiFragsNets*. The estimated exact *m/z* for protonated sulfamethoxazole (C_10_SO_3_N_3_H_12_^+^) is 254.0599374277 Da. Table 1 contains the conventional information for the MS2 sulfamethoxazole spectrum in the columns “Meassured *m/z* (Da)” and “Relative intensity (%)”, but also the uncertainty for each *m/z* in the “Confidence interval (ppm)” column, and the number of signals clustered on each peak in the “#Data points” column. The annotation for each fragment is in the column “Molecular formula”, the corresponding *m/z* for that annotation in “Predicted *m/z* (Da)”, and the mass error, between the predicted *m/z* and the measured *m/z*, in the column “Mass error (ppm)”.

**Table 1:** Example of HRMS automatic annotation by *OrbiFragsNets* for sulfamethoxazole.

Molecular formula	Mass error (ppm)	Predicted *m/z* (Da)	Measured *m/z* (Da)	Confidence interval (ppm)	Relative intensity (%)	#Data points
C_6_NH_6_^+^	0.2	92.04947562	92.04945365	22	7.1	8
C_6_NH_7_^+^	0.6	93.05730065	93.05724538	23	5.6	8
C_4_ON_2_H_7_^+^	0.9	99.05528927	99.05519608	26	6.2	7
C_6_ONH_6_^+^	1.4	108.0443902	108.0442397	24	10.3	8
C_8_N_3_H_9_^+^	0.3	147.0790987	147.0790478	28	4.8	8
C_8_N_3_H_10_^+^	1.2	148.0869238	148.0867458	31	1.6	7
C_6_SO_2_NH_6_^+^	0.6	156.011376	156.0112839	30	44.1	8
C_9_O_2_NH_3_^+^	9.5	157.0158298	157.0143411	30	1.6	8
C_9_N_3_H_10_^+^	0.5	160.0869238	160.0868473	30	3.3	8
C_10_ON_3_H_10_^+^	0.4	188.0818384	188.0817591	32	5.6	8
C_10_SO_3_N_3_H_12_^+^	0.5	254.0593888	254.059274	38	9.8	8

### Testing *orbifragsnets*

Tables 2, 3, and 4 include the *m/z* values taken from spectra for carbamazepine, sulfamethoxazole, and atenolol, respectively, as well as their corresponding annotations by *OrbiFragsNets*, RMassBank [16] and SIRIUS [18]. To make a fair comparison between the three software tools, we took the already annotated spectra from the MassBank and annotated with *OrbiFragsNets* and SIRIUS [18]. For the annotation we only considered the fragments whose contribution to the total intensity was higher than 1%.

**Table 2:** Comparison of automatic annotation for carbamazepine MS2 spectrum by *OrbiFragsNets*, RMassBank, and SIRIUS. The original spectrum was taken from the MassBank (https://massbank.eu/MassBank/RecordDisplay?id=MSBNK-Athens_Univ-AU112001).

Measured *m/z* (Da)	OrbiFragsNets	RMassBank	SIRIUS
194.0964	C_14_NH_12_^+^	C_14_NH_12_^+^	C_14_NH_12_^+^
237.102	C_15_ON_2_H_13_^+^	C_15_ON_2_H_13_^+^	C_15_ON_2_H_13_^+^
238.1052	–	C_14_^13^CON_2_H_13_^+^	–

**Table 3:** Comparison of automatic annotation for sulfamethoxazole MS2 spectrum by *OrbiFragsNets*, RMassBank, and SIRIUS. The original spectrum was taken from the MassBank (https://massbank.eu/MassBank/RecordDisplay?id=MSBNK-Athens_Univ-AU101801&dsn=Athens_Univ).

Measured *m/z* (Da)	OrbiFragsNets	RMassBank	SIRIUS
65.0382	C_5_H_5_^+^	C_5_H_5_^+^	C_5_H_5_^+^
68.049	C_4_NH_6_^+^	C_4_NH_6_^+^	C_4_NH_6_^+^
92.0496	C_6_NH_6_^+^	C_6_NH_6_^+^	C_6_NH_6_^+^
93.0575	C_6_NH_7_^+^	C_6_NH_7_^+^	C_6_NH_7_^+^
99.0562	C_4_ON_2_H_7_^+^	C_4_ON_2_H_7_^+^	C_4_ON_2_H_7_^+^
108.046	C_6_ONH_6_^+^	C_6_ONH_6_^+^	C_6_ONH_6_^+^
110.0605	C_6_ONH_8_^+^	C_6_ONH_8_^+^	C_6_ONH_8_^+^
146.071	C_8_N_3_H_8_^+^	C_8_N_3_H_8_^+^	C_8_N_3_H_8_^+^
147.0791	C_8_N_3_H_9_^+^	C_8_N_3_H_9_^+^	C_8_N_3_H_9_^+^
148.0864	C_8_N_3_H_10_^+^	C_8_N_3_H_10_^+^	C_8_N_3_H_10_^+^
156.0119	C_6_SO_2_NH_6_^+^	C_6_SO_2_NH_6_^+^	C_6_SO_2_NH_6_^+^
157.0146	–	C_9_O_2_NH_3_^+^	–
158.0078	–	C_9_SNH_4_^+^	–
160.0873	C_9_N_3_H_10_^+^	C_9_N_3_H_10_^+^	C_9_N_3_H_10_^+^
188.0822	C_10_ON_3_H_10_^+^	C_10_ON_3_H_10_^+^	C_10_ON_3_H_10_^+^
254.0603	C_10_SO_3_N_3_H_12_^+^	C_10_SO_3_N_3_H_12_^+^	C_10_SO_3_N_3_H_12_^+^

**Table 4:** Comparison of automatic annotation for atenolol MS2 spectrum by *OrbiFragsNets*, RMassBank, and SIRIUS. The original spectrum was taken from the MassBank (https://massbank.eu/MassBank/RecordDisplay?id=MSBNK-Athens_Univ-AU110601&dsn=Athens_Univ).

Measured *m/z* (Da)	OrbiFragsNets	RMassBank	SIRIUS
56.0491	C_3_NH_6_^+^	C_3_NH_6_^+^	C_3_NH_6_^+^
72.0804	C_4_NH_10_^+^	C_4_NH_10_^+^	C_4_NH_10_^+^
74.0597	C_3_ONH_8_^+^	C_3_ONH_8_^+^	C_3_ONH_8_^+^
98.0973	C_6_NH_12_^+^	C_6_NH_12_^+^	C_6_NH_12_^+^
116.1079	C_6_ONH_14_^+^	C_6_ONH_14_^+^	C_6_ONH_14_^+^
133.0651	C_9_OH_9_^+^	C_9_OH_9_^+^	C_9_OH_9_^+^
145.0648	C_10_OH_9_^+^	C_10_OH_9_^+^	C_10_OH_9_^+^
162.0914	C_10_ONH_12_^+^	C_10_ONH_12_^+^	C_10_ONH_12_^+^
164.0705	C_9_O_2_NH_10_^+^	C_9_O_2_NH_10_^+^	C_9_O_2_NH_10_^+^
173.0597	C_11_O_2_H_9_^+^	C_11_O_2_H_9_^+^	C_11_O_2_H_9_^+^
178.0867	C_10_O_2_NH_12_^+^	C_10_O_2_NH_12_^+^	C_10_O_2_NH_12_^+^
180.1022	C_10_O_2_NH_14_^+^	C_10_O_2_NH_14_^+^	C_10_O_2_NH_14_^+^
190.0866	C_11_O_2_NH_12_^+^	C_11_O_2_NH_12_^+^	C_11_O_2_NH_12_^+^
191.0898	–	C_11_O_2_NH_13_^+^	–
208.0976	C_11_O_3_NH_14_^+^	C_11_O_3_NH_14_^+^	C_11_O_3_NH_14_^+^
225.1239	C_11_O_3_N_2_H_17_^+^	C_11_O_3_N_2_H_17_^+^	C_11_O_3_N_2_H_17_^+^
267.1709	C_14_O_3_N_2_H_23_^+^	C_14_O_3_N_2_H_23_^+^	C_14_O_3_N_2_H_23_^+^
268.1747	–	C_13_O_3_N_3_H_22_^+^	–

It is important to mention that using already centroided data undervalue the lack of pre-processing and the estimation of confidence interval for specific fragments in specific matrixes in *OrbiFragsNets. OrbiFragsNets* estimates the confidence interval for each peak, but for comparing already pre-processed data we assign a value of 10 ppm, in *OrbiFragsNets* and SIRIUS. The notebook ‘*OrbiFragsNets_example.ipynb*’ contains a script for the annotation.

Annotated fragments in Table 2, Table 3, Table 4 show that our offline tool *OrbiFragsNets* can perform as good as SIRIUS. The few fragments only annotated by RMassBank, are not part of the fragmentation process according to SIRIUS, and similarly not consistent with the other fragments according to *OrbiFragsNets*. The GitHub library is constantly updated and will provide more examples of annotation and comparison with other tools.

The next step in developing this methodology is a mathematical formulation of the chemical consistency as a hypothesis and a statistical analysis to connect the grade of our fragments networks with the accuracy of the predictions.

The annotation of the MS2 spectra could become the first step in the data processing for identification of unknown compounds. Automatic annotation of MS2 spectra increases the confidence in the alignment of features when comparing samples, as there is already certainty on the identity of the feature, furthermore, the confidence in approaches such as molecular networking would increase, as no mass error is needed when the spectrum can be aligned directly.

Spectra annotation as a first step in data processing could an be an advantage over conventional workflows, and our approach offers the basics for a *de novo* workflow. Chemical consistency is independent of any external data and therefore is valid for unknown substances, too. Self-consistent annotated spectra also offer an alternative to input data for structural elucidation using machine learning.

## CRediT authorship contribution statement

**Edwin Chingate:** Conceptualization, Methodology, Software, Writing – original draft, Writing – review & editing, Visualization. **Jörg E. Drewes:** Writing – review & editing, Supervision, Funding acquisition. **María José Farré:** Conceptualization, Writing – original draft, Writing – review & editing, Supervision, Funding acquisition. **Uwe Hübner:** Conceptualization, Writing – original draft, Writing – review & editing, Supervision, Funding acquisition.

## Declaration of Competing Interest

The authors declare that they have no known competing financial interests or personal relationships that could have appeared to influence the work reported in this paper.

## Appendix Supplementary materials

Image, application 1
